# A Modified EMD Technique for Broken Rotor Bar Fault Detection in Induction Machines

**DOI:** 10.3390/s24165186

**Published:** 2024-08-11

**Authors:** Md. Shamsul Arifin, Wilson Wang, Mohammad Nasir Uddin

**Affiliations:** 1 Department of Electrical and Computer Engineering, Lakehead University, GC Campus, Barrie, ON L4M 3X9, Canada; arifinm@lakeheadu.ca (M.S.A.); muddin@lakeheadu.ca (M.N.U.); 2 Department of Mechanical and Mechatronics Engineering, Lakehead University, Thunder Bay, ON P7B 5E1, Canada

**Keywords:** induction machines, rotor bar fault detection, smart current sensors, motor current signature analysis, empirical mode decomposition, spectral operation

## Abstract

Induction machines (IMs) are commonly used in various industrial sectors. It is essential to recognize IM defects at their earliest stage so as to prevent machine performance degradation and improve production quality and safety. This work will focus on IM broken rotor bar (BRB) fault detection, as BRB fault could generate extra heating, vibration, acoustic noise, or even sparks in IMs. In this paper, a modified empirical mode decomposition (EMD) technique, or MEMD, is proposed for BRB fault detection using motor current signature analysis. A smart sensor-based data acquisition (DAQ) system is developed by our research team and is used to collect current signals wirelessly. The MEMD takes several processing steps. Firstly, correlation-based EMD analysis is undertaken to select the most representative intrinsic mode function (IMF). Secondly, an adaptive window function is suggested for spectral operation and analysis to detect the BRB fault. Thirdly, a new reference function is proposed to generate the fault index for fault severity diagnosis analytically. The effectiveness of the proposed MEMD technique is verified experimentally.

## 1. Introduction

Induction machines (IMs) have become a powerhouse in various industrial sectors such as electric vehicles, manufacturing, and power generation. Based on investigation, up to 60% of the electric energy generated in the world is consumed by IMs. However, IM defects can degrade operation performance and quality of the related machines. These IM faults can be induced by electrical, thermal, or mechanical stress. The faults commonly found in IMs are the stator winding defects, bearing damage, broken rotor bar (BRB) faults, etc. [1,2]. Although the BRB fault may take approximately 10% of the IM perfections, BRBs can cause extra vibration, acoustic noise, power loss, damage of adjacent rotor bars, or even sparks. Hence, this work will focus on IM BRB fault detection and diagnosis.

Several types of physical signals can be applied for IM condition monitoring and BRB fault detection, such as magnetic flux, vibration, electric current, voltage, etc. However, motor current signature analysis (MCSA) is commonly used due to its relative ease of measurement and higher signal-to-noise ratio for BRB fault detection [1,3]. This work will also be based on motor current signal analysis. A smart current sensor network and data acquisition (DAQ) system have been developed by our research team and are used for current data collection wirelessly in this work.

A number of signal processing techniques are applied for BRB fault detection of IMs. Some of the mostly used techniques are the fast Fourier transform (FT), short-time FT, discrete and continuous wavelet transform (WT), Hilbert–Huang transform (HHT), etc. [1,3]. For example, BRB fault is detected using an FT technique using stator flux in [4], a short-time FT analysis in [4], and power spectral density processing in [5]. In addition, recent studies also consider the torque and speed spectra of IMs for FT analysis to detect BRB faults [6]. However, the classical FT-based spectral analysis is not suitable for nonstationary signal analysis, whereas signatures from IMs with BRB faults are usually time-varying and nonstationary. Consequently, several time-frequency domain techniques have been proposed in the literature to detect the BRB fault in IMs [7,8]. For example, a wavelet packet transform-based HHT technique is presented in [7], and an HHT along with neural networks technique is used in [8] to detect BRB fault in IMs. The WT using DB-27 from Daubechies wavelet family is used in [9] to analyze and detect the BRB fault from MCSA. Additionally, some studies also utilize empirical mode decomposition (EMD) to detect BRB fault of IMs [1,10,11,12]. For example, a classical EMD method is used in [12] to analyze BRB faults; however, this study only considers the amplitudes of selected IMFs for BRB fault detection without providing theoretical explanation for IMF selection. Additionally, no quantitative analysis or index is provided to indicate the fault severity. On the other hand, a number of recent studies used the variational mode decomposition (VMD) technique and its modified versions to detect the BRB faults [13,14,15,16]. For example, a modified VMD method is presented in [13] to detect BRB fault; however, the analysis of fault severity is not provided. In [14], a classical VMD method along with the machine learning is presented to predict different number of BRBs; however, the study does not clarify how to select the best classifier for BRB fault diagnosis. Paper [15] presents a VMD method for detecting wound rotor defects in IMs. However, this study focuses on processing a specific IMF (i.e., IMF_5_), which may not be robust for different types of motors and variable operating conditions. Furthermore, a fuzzy neural network and polar image-based VMD technique is presented in [16]. However, the technique could suffer from extra computation burden due to the use of several processing units and the results could be difficult to explain due to black-box-related training.

A number of researches have been conducted to detect the BRB fault in the IM by applying some other signal processing techniques [17,18,19,20,21,22,23,24,25,26]. For example, a 3D sensor-based signal acquisition and analysis technique is presented in [22] by analyzing emf induced in these sensor coils in radial and axial positions of the IM; however, the study does not provide sensor position clarification properly. In [23], the sum of the weighted Fourier coefficients is applied to detect the BRB fault; however, it does not consider the impact factors related to the number and the orientation of broken bars in analysis. A rotational invariance processing method is suggested in [24] to detect the BRB fault considering the oscillations due to IM load variations. The oscillations due to load variations are dependent of several factors such as nature of the load, level of load variations, and power supply variations; however, the effectiveness of that technique is not verified comprehensively corresponding to different IM conditions. In addition, some studies have used neural networks [2,8], fuzzy reasoning [6], and machine learning methods [5,27,28] to detect IM BRB fault. However, clarification of the processing results using these supervised training algorithms may be difficult, especially under variable motor operating conditions in terms of load and speed. A genetic algorithm-based method is proposed in [28] to detect the BRB fault. However, the genetic algorithm suffers from high computational burden and some uncertainty to select the optimum result.

From the systematic literature review, it is observed that most of the studies have focused on detecting the BRB fault considering different signatures obtained by applying different signal processing methods and machine learning algorithms. However, very little research has considered to estimate a quantitative fault index to predict the health states and fault severity of the IMs. In order to further improve the accuracy of signal processing-based IM BRB fault detection, a modified EMD (MEMD) technique is proposed in this paper to recognize representative features for IM fault detection, specifically for BRB fault detection, based on analysis of current signals collected using our developed smart current sensors. The proposed MEMD technique is new in the following aspects: (1) an adaptive window spectral operation (AWSO) technique is proposed to recognize the sidebands of the characteristic frequency of the selected intrinsic mode function (IMF) through the EMD processing. The AWSO can adjust its bandwidth adaptively without reconstructing the original time domain signal. (2) A new post-processing method is suggested to generate the fault index for the BRB fault diagnosis and severity assessment. The effectiveness of the proposed MEMD technique is verified experimentally.

The rest of this paper is organized as follows. The proposed MEMD technique is presented in Section 2. The effectiveness of the proposed MEMD technique is verified using experimental data in Section 3.

## 2. The Proposed MEMD Technique for BRB Fault Detection

**Figure 1:**
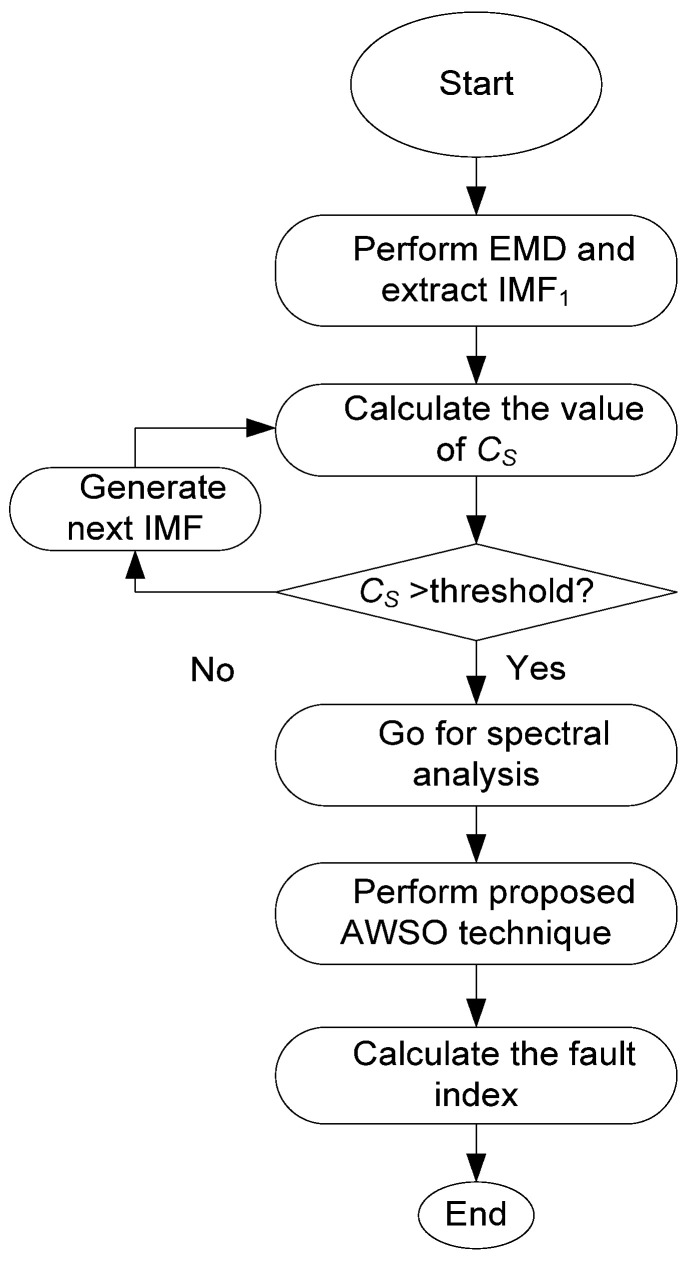
The operational steps of the MEMD technique.

The proposed MEMD technique uses three processing procedures in analysis: firstly, an adaptive EMD approach is suggested to select the most representative IMF, or IMF_1_, which will be used for analysis to facilitate processing for real-time IM condition monitoring. Secondly, an AWSO method is applied to adaptively extract the representative features from the IMF spectrum. Thirdly, the post-processing method is applied to generate the fault index for BRB fault detection and severity diagnosis. The processing procedures are summarized in Figure 1. Details will be discussed in the following subsections.

### 2.1. The Adaptive EMD Technique for IMF Selection

The EMD is a technique to decompose a signal *x*(*t*) into several IMFs, or

(1) $$x\left(t\right)=\sum_{i=1}^{N}y_{i}\left(t\right)+r\left(t\right)$$

where $y_{i}\left(t\right)$ is the *i*th IMF and *r*(*t*) is the residue. The process of producing an IMF starts by calculating the mean value $\bar{x}$ as

(2) $$\bar{x}\left(t\right)=\frac{E_{max}+E_{min}}{2}$$

where *E_max_* and *E_min_* are the maximum and minimum values of the locally developed envelope, respectively. The intermediate signal $g_{1}$ is generated as

(3) $$g_{1}=x\left(t\right)-\bar{x}\left(t\right)$$

This $g_{1}$ can be considered as an IMF only if it can fulfil the following two conditions:

(1) The difference between the number of extrema and the number of zero-crossing points is either 0 or 1.
(2) The mean value of the envelope due to the local maxima and local minima is zero.

If $g_{1}$ cannot fulfill these criteria for an IMF, then the original signal *x*(*t*) is replaced by $g_{1}$ and the same processes are repeated until the IMF_1_ is formulated (or IMF_1_ := $g_{1}$). Then the residual will be calculated as

(4) $${r\left(t\right)=x\left(t\right)-g}_{1}$$

$r\left(t\right)$ will be used as an input signal to calculate the following IMFs. The procedures are followed until the desired number of IMFs are formulated [11].

After the first IMF or IMF_1_ is generated, it is further compared with an ideal benchmark signal that has the same frequency components as the operating frequency of the power line with which the IM is connected. The correlation coefficient *C_S_* is computed to estimate this similarity, which can be calculated as

(5) $$C_{S}=\frac{\sum_{l=-N}^{N}\sum_{n=0}^{N-1}S_{1}\left(n\right)\times S_{2}\left(n-l\right)}{\sqrt{\left[\sum_{l=-N}^{N}\sum_{n=0}^{N-1}S_{1}\left(n\right)\times S_{1}\left(n-l\right)\right]\times \left[\sum_{l=-N}^{N}\sum_{n=0}^{N-1}S_{2}\left(n\right)\times S_{2}\left(n-l\right)\right]}}$$

where $S_{1}$ and $S_{2}$ are two signals to be compared or the IMF_1_ and the benchmark signal, *N* = the length of signal. If the value of *C_S_* is greater than a threshold, the IMF_1_ will be considered the most representative feature, and will be used for the following spectral analysis; otherwise, another IMF will be generated and examined correspondingly. The estimation procedure of threshold of *C_S_* will be discussed in Section 3.

### 2.2. The Proposed AWSO Technique

The symmetry inside the rotor current flow will be interrupted if one or more rotor bars are broken. Due to this asymmetric rotor current flow, the magnetic field inside the IM is interrupted, which will create disturbances like overheating, vibration, etc. In addition, extra sidebands will be generated in the spectrum of the IM’s current as

(6) $$f_{ck}=f_{B}\left(1\pm 2ks\right)$$

where $f_{ck}$ is the characteristic frequency at *k*th side band, k=0, 1, 2,…; $f_{B}$ is the fundamental line frequency; *s* is the slip of the rotor calculated as $s=\frac{n_{s}-n_{r}}{n_{s}}$; *n_s_* and *n_r_* are the synchronous and the rotor speed in rpm, respectively.

The proposed AWSO method applies adaptive windows to recognize the faulty features from the side band spectra of MCSA. This proposed adaptive window can adjust its bandwidth $B_{ck}$ based on the IM speed (i.e., slip *s*) and the order of sideband (*k*), such that

(7) $$B_{ck}=\frac{s\times \eta }{2^{\left(k-1\right)}}$$

where the value of η depends on IM structures and operating conditions (i.e., speed or slip). The selection of the value of η will be discussed in Section 3.3. Hence, the lower and upper frequency range ($f_{ck-L},\;f_{ck-U}$) of the window function can be calculated as

(8) $$f_{ck-U}=f_{ck}+B_{ck}$$

(9) $$f_{ck-L}=f_{ck}-B_{ck}$$

Thus, the proposed adaptive window function *W_ck_* can be represented as

(10) $$W_{\mathrm{ck}}={\;a}_{c}\left({\left[1+e^{-h\left(f-f_{ck-L}\right)}\right]}^{-1}-{\left[1+e^{-h\left(f-f_{ck-U}\right)}\right]}^{-1}\right)$$

where $a_{c}$ is a parameter that can accentuate the spectral components, and *h* represents a parameter related to the sigmoid function [29] used to formulate *W_ck_*.

**Figure 2:**
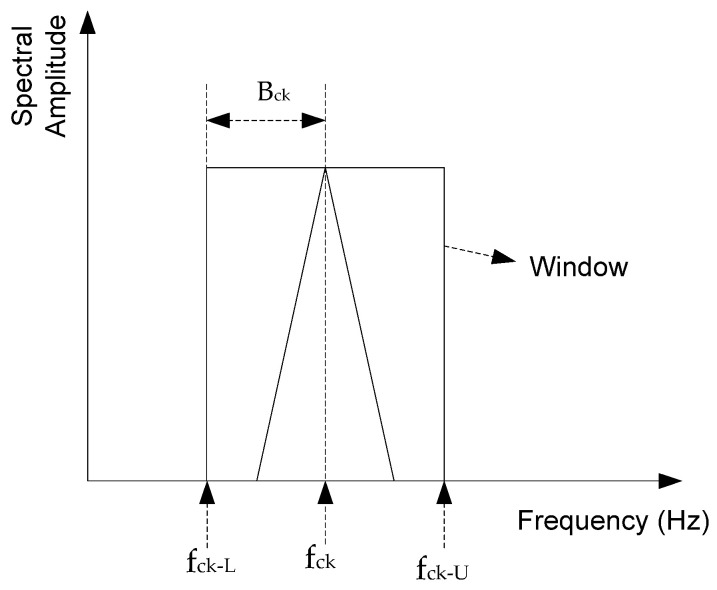
The graphical representation of the proposed adaptive window function.

Figure 2 illustrates the adaptive window function graphically.

The spectral analysis of the motor current signal generates the dataset (*T_S_*) that contains a combination of data having spectral values *y_m_* at corresponding frequency *f_m_*, which can be mathematically represented as

(11) $$T_{S}=\underset{m}{⋃}\left(y_{m},\;f_{m}\right)$$

where $T_{S}\;\epsilon \;R^{2}\;contains$ two-dimensional data; m ϵ N is a natural number, N={1, 2, 3…}; and the union operator U is to combine and highlight the data points in dataset *T_S_*.

The dataset *T_B_* is formulated by introducing the data adjacent to the basic line frequency *f_B_*, such that

(12) $$T_{B}=\underset{m_{1}}{⋃}\left(y_{m_{1}},\;f_{m_{1}}\right)$$

where $T_{B}\epsilon R^{2},\;m_{1}\epsilon N$; $y_{m_{1}}$ is the spectral value of frequency $f_{m_{1}}$ on $T_{B}$.

The following spectral operation is performed to construct *D_B_* using

(13) $$y_{m_{1}}≔y_{m},\;f_{m_{1}}\epsilon \left[f_{B}-\varepsilon ,\;f_{B}+\varepsilon \right]$$

where the value of ε depends on IM structures and operating conditions, which will be discussed in Section 3.3. Additionally, datasets *T_ck_* are formulated based on the sideband frequencies, such that

(14) $$T_{ck}=\underset{m_{2}}{⋃}\left(y_{m_{2}},f_{m_{2}}\right)$$

where $T_{ck}\epsilon R^{2}\;and\;m_{2}\epsilon N$.

The proposed AWSO method is applied on the data of $T_{ck}$, or

(15) $$y_{m_{2}}≔{y_{n}W}_{ck},\;f_{m_{2}}\epsilon \left[f_{ck-L},\;f_{ck-U}\right]$$

The dataset *T_rk_* that does not incorporate either $f_{ck}$ or $f_{B}$ can be formulated as

(16) $$T_{rk}=\underset{m_{3}}{⋃}\left(y_{m_{3}},\;f_{m_{3}}\right)$$

where $T_{rk\pm }\epsilon R^{2}\;and\;m_{3}\epsilon N$.

A moving average operation is undertaken on the data $T_{rk}$, such that

(17) $$y_{m3}≔\frac{1}{n}\sum_{i=0}^{n-1}y_{n-i},\;f_{m3}\epsilon \left[f_{c\left(k-1\right)-U},\;f_{ck-L}\right]$$

Finally, a modified spectral dataset *T_M_* is created by combining all the processed data of different dataset, such that

(18) $$T_{M}=T_{B}\underset{k}{⋁}T_{ck}\underset{k}{⋁}T_{rk}$$

where V operator represents the integration operation of the datasets. The operation steps of AWSO method are summarized below:

Step 1: Determine $f_{ck}$ and $B_{ck}$ using Equations (6) and (7).

Step 2: Compute the frequency ranges $f_{ck-U,}{\;f}_{ck-L}$ using Equations (8) and (9).

Step 3: Construct adaptive window *W_ck_* using Equation (10).

Step 4: Construct the datasets *T_B_, T_ck_,* and *T_rk_* using Equations (11)–(17).

Step 5: Formulate *T_M_* using Equation (18).

### 2.3. The Proposed Post-Processing Function

A new post-processing function $F_{P}$ is suggested to generate the fault index for fault severity assessment:

(19) $$F_{P}=\sum_{\begin{array}{c} k=-n\\ k\neq 0 \end{array}}^{n}r_{k}\frac{y_{ck}-y_{avg,k}}{y_{B}-y_{ck}}$$

where $y_{ck}$ is the spectral value at $f_{ck}$; $y_{B}$ is the spectral value at $f_{B}$; $y_{avg,k}$ is average spectral value associated with $f_{ck}$; and $r_{k}$ is the scaling factor. The selection of $r_{k}$ is discussed in Section 3.

A higher index value of $F_{P}$ indicates more severity of fault in IM and vice versa.

In comparison, the commonly used fault index function *F_I_* in the literature [10,24] is presented in Equation (20). From our experience, *F_I_* is not very robust in many applications.

(20) $$F_{I}=\sum_{\begin{array}{c} k=-n\\ k\neq 0 \end{array}}^{n}\frac{y_{ck}-y_{avg,k}}{y_{avg,k}}$$

## 3. Experimental Tests and Performance Analysis

The effectiveness of the proposed MEMD technique is verified in this section. Firstly, the experimental setup and smart current sensor-based DAQ system are described. Secondly, the related parameters selection criteria are discussed, followed by experimental tests of the proposed MEMD technique under different IM operating conditions.

### 3.1. The Smart Current Sensors DAQ System

**Figure 3:**
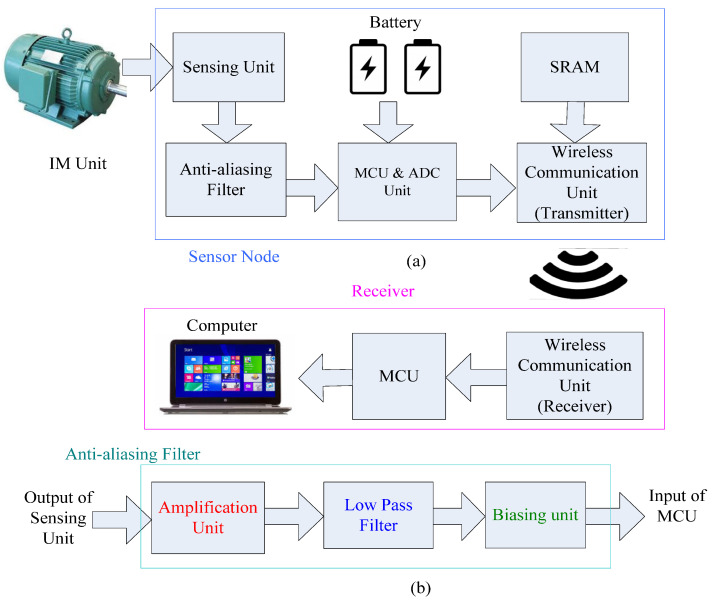
The structure of: (**a**) the smart DAQ, (**b**) anti-aliasing filter.

A smart current sensor is developed by our research team for DAQ. The smart current sensors-based DAQ system consists of operational modules such as sensing units, signal pre-conditioning unit, microcontroller unit (MCU), analog-to-digital converter (ADC), wireless communication units, and computer interfacing equipment. The structure of the DAQ system is illustrated in Figure 3a.

The current sensing unit of the DAQ primarily uses a split-core current transformer C_CT 16 (Nidec Copal Electronics, Tokyo, Japan). The current signals collected by the sensing units are preprocessed by a signal-conditioning unit, which has several functions like amplifier, antialiasing filter, biasing circuits, etc., as illustrated in Figure 3b. The selected MCU is PIC32MX250F128B (Microchip Technology, Chandler, AZ, USA) with reprogrammable ADC pins. The wireless transceiver chip CC1101 (Texas Instruments, Dallas, TX, USA) is utilized for wireless communication. In this smart sensor-based DAQ, the 915-MHz ultra-high frequency band is selected for communication. The receiver unit receives data from different sensor nodes through wireless communication medium [30]. This receiver is interfaced with a computer in which the digital data are further processed using advanced signal processing techniques, including the proposed MEMD for IM condition monitoring and BRB fault detection.

### 3.2. Experimental Setup

**Figure 4:**
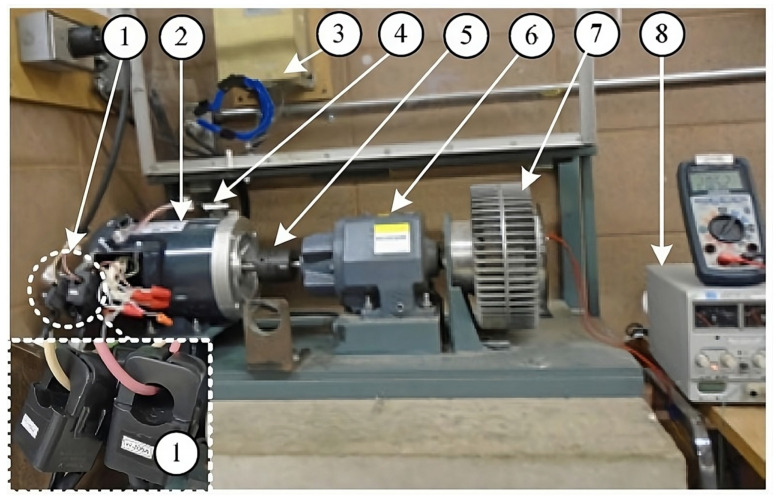
Experimental setup: (1) smart current sensors; (2) IM; (3) power supply with VFD; (4) vibration sensor; (5) elastic coupling; (6) gearbox; (7) magnetic clutch; (8) DC supply, and (9) enclosure.

Figure 4 shows the experimental setup used in this work. Several IMs have been tested with conditions such as healthy IMs and faulty IMs with different number of BRBs to simulate different fault severity. The tested motors (model 056T34F5301 from Marathon Electric) are rated at 0.372 KW (1/2 HP), which have 24 and 34 bars in the stator and rotor, respectively. A variable frequency drive (VFD) has a modulated frequency of 15 KHz and is used to alter the line frequency between 50 Hz and 60 Hz. A magnetic clutch is used to change the loading condition of IMs. The gearbox is used to adjust the motor speed over the operation range of the magnetic clutch. The line current data are collected at a sampling frequency of 1.0 KHz using the developed smart current sensors DAQ system as discussed earlier, with four load conditions as summarized in Table 1.

### 3.3. MEMD Parameter Selection

**Figure 5:**
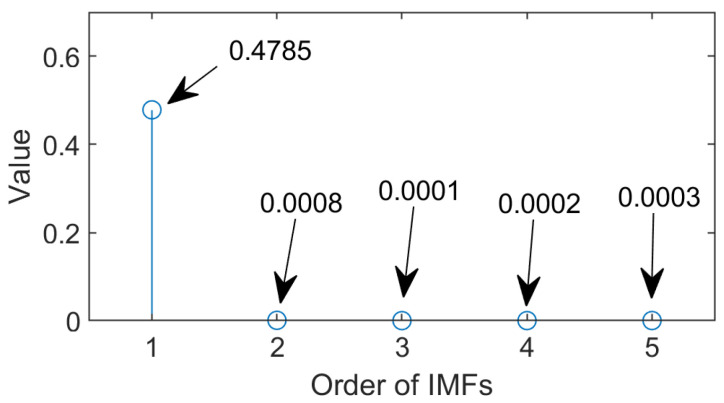
The values of *C_S_* for the first five IMFs for an IM with the slip of 2.2% and three BRBs.

To implement the proposed MEMD technique, it is necessary to choose the threshold value of *C_S_* in Equation (5) as discussed in Section 2.1. To estimate the threshold value of *C_S_* for the IMFs, systematic tests have been undertaken under different operating conditions, as indicated in Table 1. Figure 5 shows an example of the values of *C_S_* for the first five IMFs for an IM with the slip of 2.2%. It is seen that the value of *C_S_* is maximum for IMF_1_ in each case, and the values of *C_S_* are negligible for other IMFs. It means that IMF_1_ contains the most representative features, which has incorporated the fundamental line frequency, the corresponding sidebands, and their associated energy. The threshold values of *C_S_* could be over the range of [0.15, 0.45], and *C_S_* = 0.4 is selected in this work.

The separation between two adjacent sidebands can be calculated using Equation (6) by setting $k=k_{1}$ and $k=k_{1}+1$, or

(21) $$f_{c\left(k1+1\right)}-f_{ck1}=f_{B}\left(1+2\left(k_{1}+1\right)s\right)-f_{B}\left(1+2k_{1}s\right)=2sf_{B}$$

From Equation (21), it is found that the separation between two adjacent sidebands depends on the slip of the IMs. Hence, the separation is minimum at no load when the slip is minimum. Similarly, the separation is maximum at full load with the maximum slip of the IM. Correspondingly, the calculated minimum and maximum separations between two adjacent sidebands are 0.48 Hz and 3.36 Hz, respectively, in this study, using Equation (6). Hence, the value of constant η $can\;be\;over\left[20,40\right]\;in\;this\;test,$ considering two counter effect states: (i) a lower value of η makes *B_ck_* in Equation (7) smaller and makes the sideband spectra narrower, which makes it difficult to extract the representative features from the spectrum; (ii) a higher value of η causes wider spectral sidebands, which may cause band overlapping in the spectra and degrade processing accuracy. η=30 is selected in this test, which provides $B_{ck}=0.12$ Hz for no load and $B_{ck}=0.84$ Hz at full load using Equation (7) for the first side band (i.e., *k* = 1). Hence, the spread of the window function for *k* = 1 can be estimated from Equations (8) and (9) as ${2\times B}_{ck}$, which is 0.24 Hz and 1.68 Hz for no load and full load, respectively, which is less than the minimum and maximum separations between two adjacent sidebands (0.48 Hz and 3.36 Hz). Therefore, no band overlapping in the spectra will happen in this study.

From systematic simulation tests, the value of ε at Equation (13) can be over [0.1, 0.2]. ε = 0.12 Hz is selected in this case, which is 25% of the minimum separation of two adjacent frequencies (0.48 Hz) to avoid band overlapping between the fundamental frequency band and its adjacent side band.

From systematic simulation tests, the value of $a_{c}$ can be over [1,2] for the tested IMs. $a_{c}$ = 1.2 is selected in this case considering the following tradeoff: the higher $a_{c}$ can result in higher spectral values from sidebands, which may overlap the spectral components at fundamental frequency and generate inaccurate prediction of severity of BRB faults by Equation (19); however, lower or no accentuation (i.e., $a_{c}$ = 1) may result in inaccurate detection of BRB faults.

### 3.4. Experimental Results and Analysis

**Figure 6:**
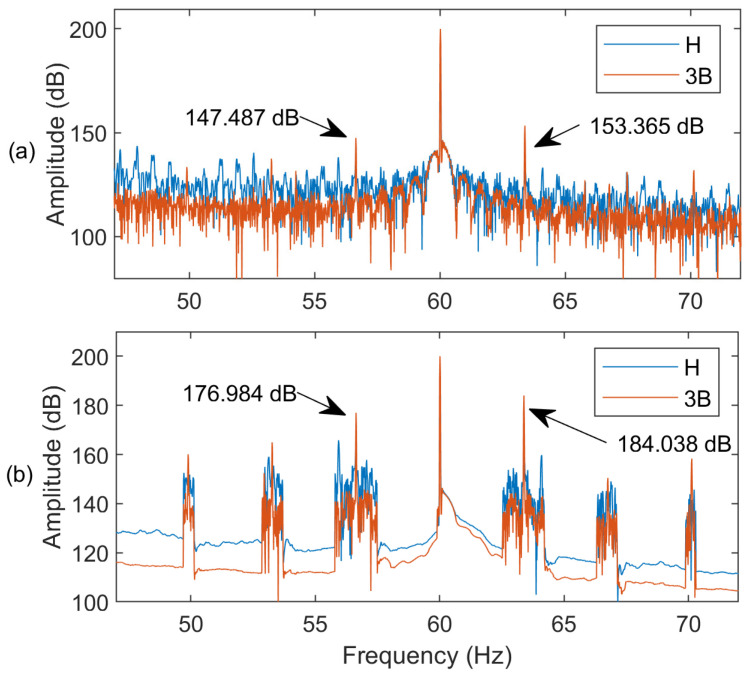
Spectra of MCSA of IM with healthy (H) and three BRBs (3B): (**a**) without applying the AWSO method; (**b**) applying the AWSO method.

Figure 6 shows the current spectra of an IM at full load (*s* = 2.8%) with and without applying the AWSO method in the MEMD technique. It is observed that the fault representative features and the characteristic side bands can be enhanced by using the proposed AWSO method.

**Figure 7:**
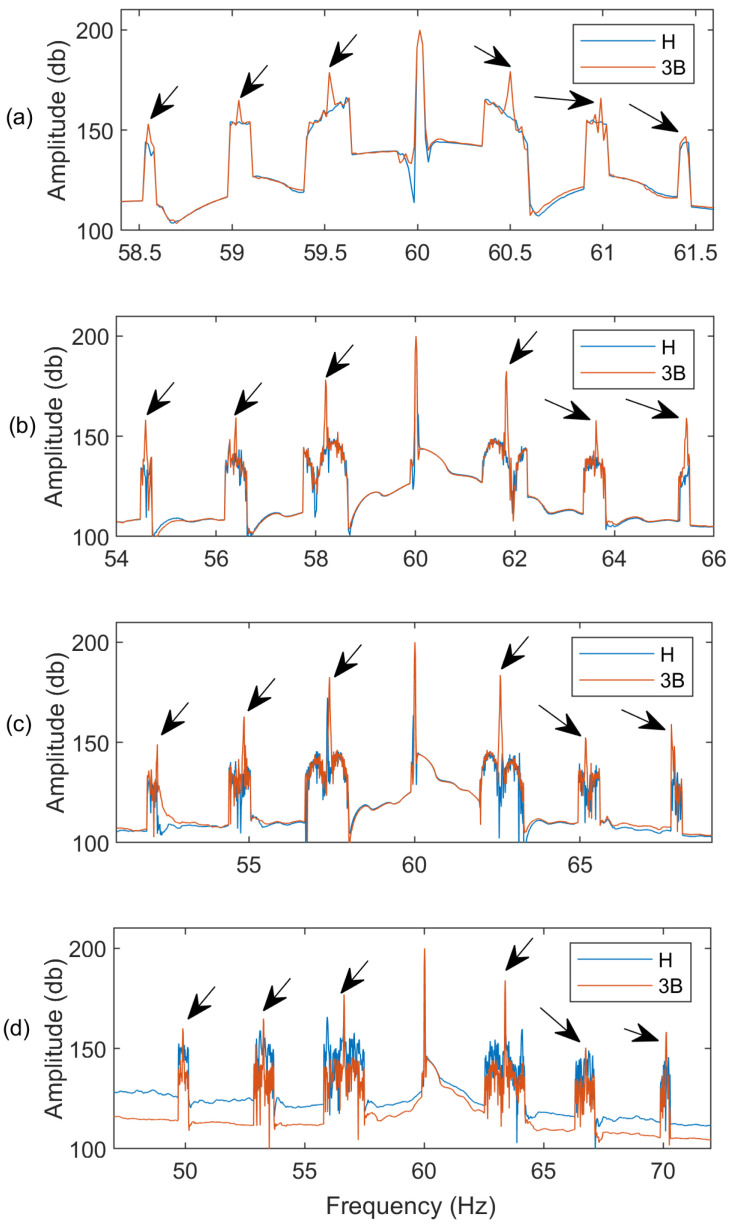
Spectra for IMs for healthy (H) and three-BRB (3B) conditions applying the AWSO technique at different speed (i.e., slip) conditions: (**a**) no load (slip = 0.4%); (**b**) light load (slip = 1.5%); (**c**) medium load (slip = 2.2%); (**d**) full load (slip = 2.8%) operating at 60-Hz line frequency.

**Figure 8:**
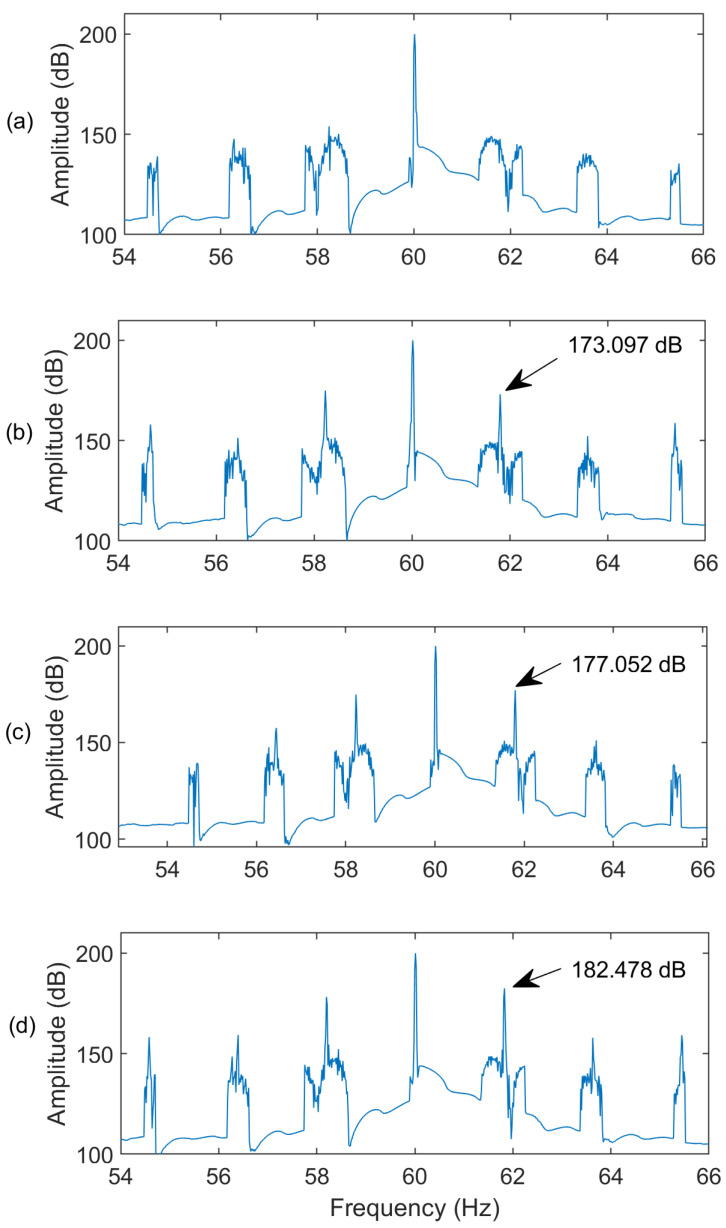
Spectra for IMs for (**a**) healthy; (**b**) one-BRB; (**c**) two-BRB; (**d**) three-BRB conditions at slip = 1.5%.

Figure 7 shows the spectra of an IM with three BRBs at different speed conditions, which are represented by different slip (*s*) levels. It is seen that the proposed MEMD technique can distinguish the BRB fault at different speed conditions due to its effective AWSO processing. Moreover, the better performance of the MEMD technique is also justified at different fault states and at different IM speed or slip conditions. Figure 8 shows the corresponding spectra of current at slip *s* = 1.5% for different IM BRB conditions. It is seen that the spectral magnitudes at the fault characteristic frequencies increase with the increasing of fault severity (i.e., the number of BRBs). Hence the proposed MEMD technique is effective to predict the fault severity, as the increase of number of BRBs will affect more operating cycles in the IM. For example, the IMs used in this experimental test have 34 rotor bars. Hence, one BRB fault affects one of 1/34 of an operating cycle, whereas three BRB faults affect three of the 1/34 of an operating cycle. Consequently, the spectral magnitudes at fault characteristic frequencies are also expected to increase with the increasing BRB severity in the IMs.

**Figure 9:**
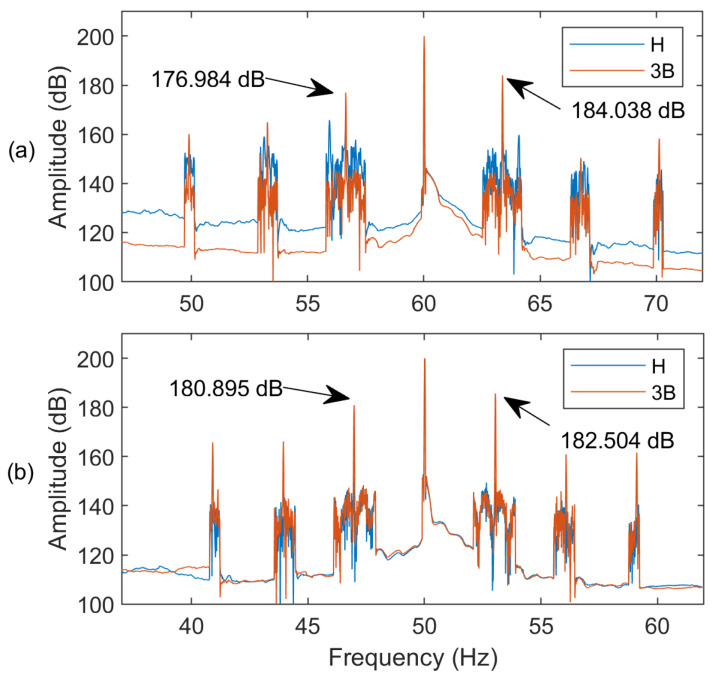
Spectra for IMs for healthy (H) and three RBR (3B) conditions applying proposed the MEMD technique (using AWSO) at (**a**) 60-Hz line frequency; (**b**) 50-Hz line frequency for full load.

Figure 9 represents the spectra for IMs with healthy and three-BRB conditions applying the proposed technique at 60-Hz and 50-Hz line frequencies for full load. It is seen that the proposed MEMD with AWSO can predict the BRB fault clearly at 50-Hz line frequency.

**Figure 10:**
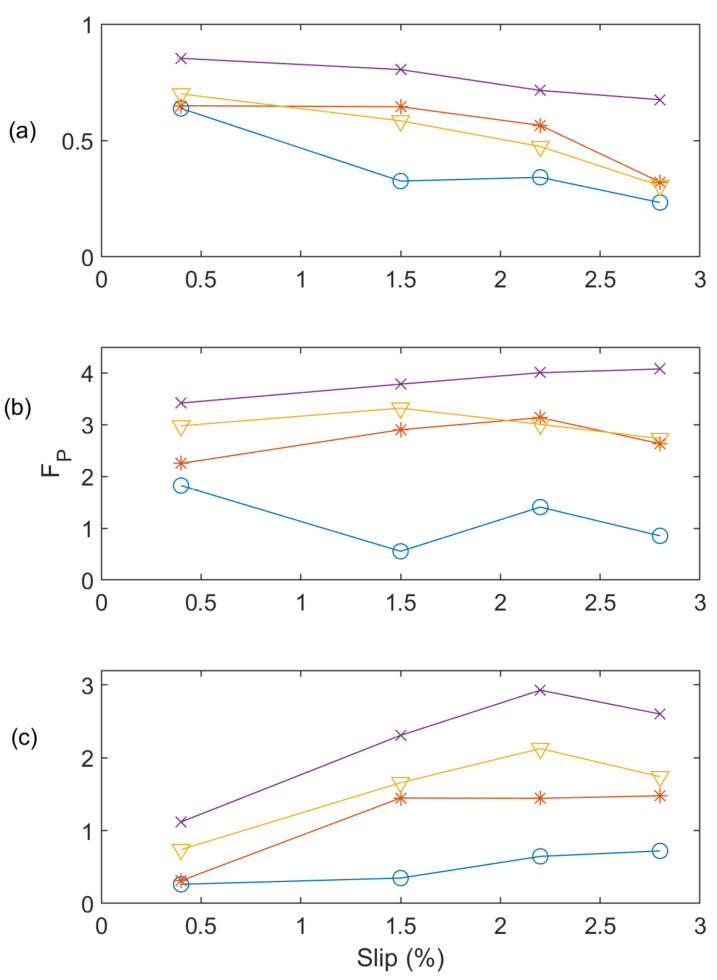
Performance comparison at 60 Hz applying related techniques (**a**) EMD-1; (**b**) EMD-2; (**c**) proposed MEMD technique under conditions of Healthy (‘O’ blue line), one BRB (‘*’ red line), two BRBs (‘∇’ yellow line), and three BRBs (‘x’ purple line).

**Figure 11:**
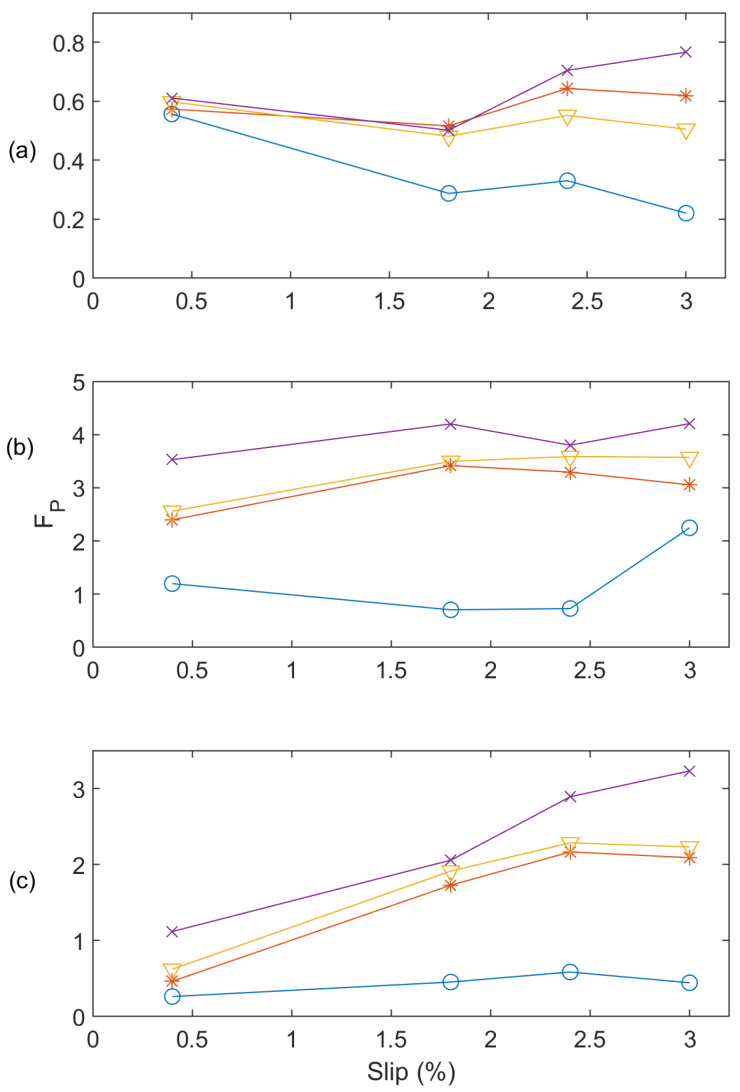
Performance comparison at 50 Hz applying related techniques (**a**) EMD-1; (**b**) EMD-2; (**c**) proposed MEMD technique for Healthy (‘O’ blue line), one BRB (‘*’ red line), two BRBs (‘∇’ yellow line), and three BRBs (‘x’ purple line).

The effectiveness of the proposed MEMD technique will be investigated in comparison with two related techniques in the literature. The first one is the classical EMD technique denoted as EMD-1, which applies no spectral operations on IMF_1_. The second is an EMD technique from [10], denoted as EMD-2, which uses variance-based spectral processing of IMF_1_. Figure 10 shows the performance comparison of the related techniques in terms of the post-processing function (*F_P_* versus *F_I_*) at different loading conditions while operating at 60 Hz. The higher values of *F_P_* and *F_I_* will specify more severe faults and vice versa. Analyzing Figure 10a, it is clear that the EMD-1 cannot distinguish between one BRB and two BRB faults at low load and medium and full load conditions. Similarly, EMD-2 cannot separate one BRB and two BRB faults clearly at medium and full load (Figure 10b). On the other hand, the proposed MEMD technique can distinguish all types of faults clearly at different load conditions as shown in Figure 10c. Figure 11 shows the performance comparison among these techniques operating at 50 Hz. It is seen that EMD-1 cannot differentiate the faults of one BRB, two BRBs and three BRBs at low load, as well as one BRB and two BRBs at medium load state. EMD-2 cannot detect the one BRB and two BRB faults clearly at low load (Figure 11b). On the contrary, as shown in Figure 11c, the proposed MEMD technique with AWSO can clearly distinguish the faults and severity condition.

In addition, the computational efficiency of the proposed MEMD technique has been analyzed in terms of time required for IMF generation, in comparison to the classical EMD technique. Table 2 shows the average computational time in milliseconds of these two techniques considering different IM load states and at healthy and three-BRB conditions. From Table 2 it is evident that the proposed MEMD technique is much more efficient in computation than the classical EMD technique. The classical EMD technique uses more time for computation as it continues generating IMF until a monotonic residue is achieved. The proposed MEMD technique, on the other hand, selects and uses the most representative IMF for processing, which makes it suitable for real-time IM condition monitoring and fault diagnosis.

**Figure 12:**
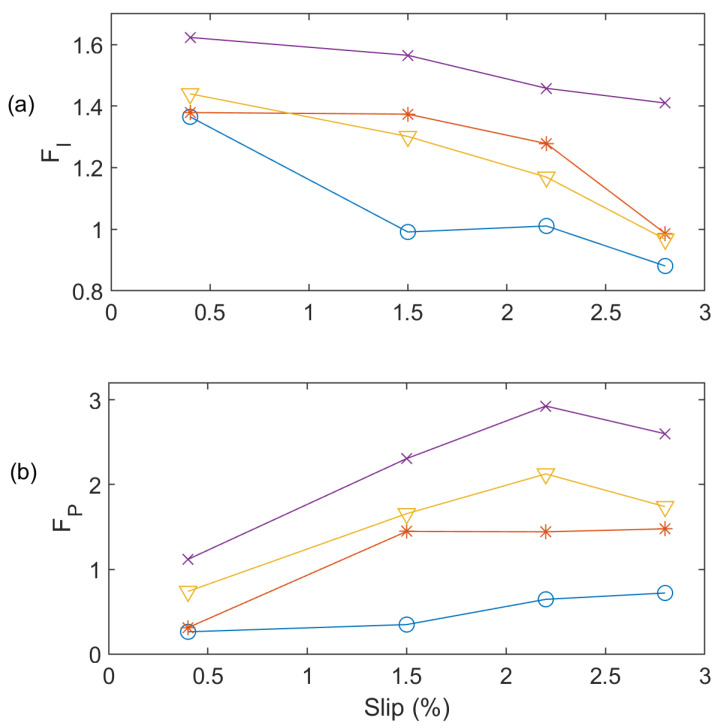
Effectiveness analysis of *F_P_* and *F_I_* at 60 Hz (**a**) *F_I_*; (**b**) *F_P_* for Healthy (‘O’ blue line), one BRB (‘*’ red line), two BRBs (‘∇’ yellow line), and three BRBs (‘x’ purple line).

**Figure 13:**
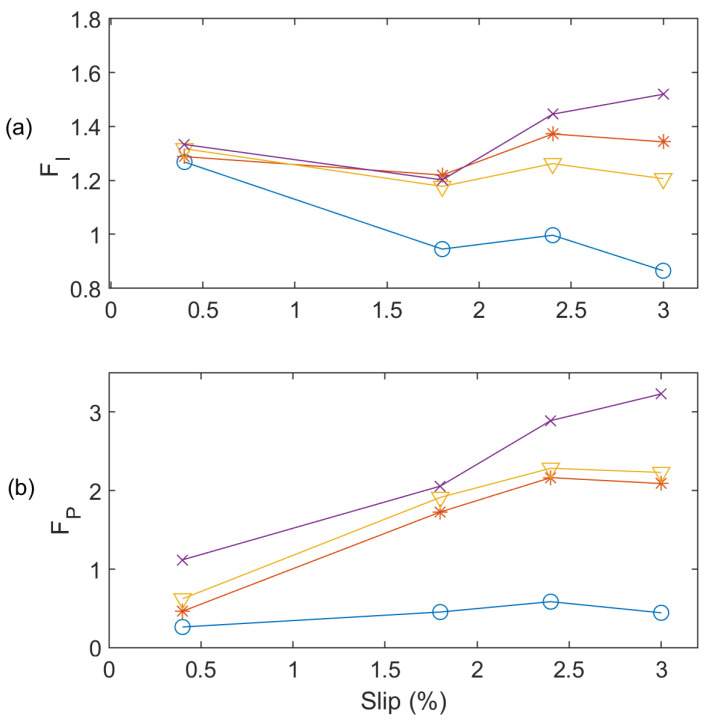
Effectiveness analysis of *F_P_* and *F_I_* at 50 Hz (**a**) *F_I_*; (**b**) *F_P_* for Healthy (‘O’ blue line), one BRB (‘*’ red line), two BRBs (‘∇’ yellow line), and three BRBs (‘x’ purple line).

The effectiveness of the *F_P_* function is also investigated in comparison with the generally used *F_I_* as given in Equation (20). Figure 12 shows the comparison of the *F_P_* versus *F_I_* at 60 Hz. It is seen that the *F_I_* cannot differentiate one BRB and two BRB faults clearly at low and full load conditions; however, the suggested *F_P_* can detect all these IM conditions clearly. On the other hand, Figure 13 shows the processing results for the test results at 50 Hz. Similarly, the *F_P_* can effectively detect faults at all loading conditions in Figure 13b whereas the *F_I_* cannot predict faulty conditions correctly at low and full load conditions (Figure 13a).

## 4. Conclusions

A modified EMD technique, or MEMD, has been proposed in this paper for BRB fault detection in IMs. A smart current sensor DAQ system is developed and used for current signal collection. The MEMD technique takes three processing steps: Firstly, a correlation-based similarity analysis is undertaken to select the targeted IMF for spectral analysis. Secondly, an AWSO method is suggested to adaptively alter its window bandwidth based on the speed and the order of the sidebands to enhance the characteristic side bands around the characteristic frequencies. With the AWSO processing, the MEMD technique does not need to reconstruct the time domain signal. Thirdly, a post-processing function *F_P_* is suggested to predict IM health conditions and assess the BRB fault severity. The effectiveness of the proposed MEMD technique has been justified experimentally corresponding to different IM rotor bar health conditions and under different operating conditions. Test results show that the proposed MEMD technique with the AWSO processing can effectively enhance characteristic features and provide more reliable BRB fault detection than the related techniques. The MEMD with the post-processing function can also predict the fault severity effectively. The proposed MEMD technique has potential to be applied in real-world IM health condition monitoring and BRB fault detection. Advanced research will be undertaken into the MEMD technique to other types of IMs and on available online data sets for BRB fault detection. Likewise, more systematic investigation will be undertaken on the effect of parameter selections in commonly used IMs. In addition, more research will be undertaken to adopt the MEMD for fault detection in other IM systems such as rolling element bearings.

## Figures and Tables

**Table 1:** IM Speed and Slip at Different Loading Conditions.

Loading	60 Hz (3600 rpm) Operation		50 Hz (3000 rpm) Operation	
	Speed (rpm)	Slip (%)	Speed (rpm)	Slip (%)
No load (0% of full load)	3586	0.4	2988	0.4
Light load (33% of full load)	3546	1.5	2946	1.8
Medium load (67% of full load)	3520	2.2	2928	2.4
Full load	3499	2.8	2910	3.0

**Table 2:** Average Computational Time for MEMD and Classical EMD Technique.

Loading Condition	HEALTH STATES	Computational Time (in Millisecond) for MEMD	Computational Time (in Millisecond) for EMD
No load (slip = 0.4%)	Healthy	6.975	48.634
	3BRB	7.648	40.07
Light load (slip = 1.5%)	Healthy	7.77	41.788
	3BRB	6.454	44.335
Medium load (slip = 2.2%)	Healthy	7.632	67.225
	3BRB	7.917	45.326
Full load (slip = 2.8%)	Healthy	9.256	53.924
	3BRB	6.674	47.742

## Data Availability

The data is unavailable.
